# Analysis of the Structure and Biosynthesis of the Lipopolysaccharide Core Oligosaccharide of *Pseudomonas syringae* pv. *tomato* DC3000

**DOI:** 10.3390/ijms22063250

**Published:** 2021-03-23

**Authors:** Alexander Kutschera, Ursula Schombel, Dominik Schwudke, Stefanie Ranf, Nicolas Gisch

**Affiliations:** 1 Chair of Phytopathology, TUM School of Life Sciences Weihenstephan, Technical University of Munich, 85354 Freising-Weihenstephan, Germany; alexander.kutschera@tum.de; 2 Division of Bioanalytical Chemistry, Priority Area Infections, Research Center Borstel, Leibniz Lung Center, Parkallee 1-40, 23845 Borstel, Germany; uschombel@fz-borstel.de (U.S.); dschwudke@fz-borstel.de (D.S.); 3 German Center for Infection Research (DZIF), 23845 Borstel, Germany; 4 Airway Research Center North, Member of the German Center for Lung Research (DZL), 23845 Borstel, Germany

**Keywords:** lipopolysaccharide, core oligosaccharide, *Pseudomonas syringae*, NMR spectroscopy, mass spectrometry, structural characterization

## Abstract

Lipopolysaccharide (LPS), the major component of the outer membrane of Gram-negative bacteria, is important for bacterial viability in general and host–pathogen interactions in particular. Negative charges at its core oligosaccharide (core-OS) contribute to membrane integrity through bridging interactions with divalent cations. The molecular structure and synthesis of the core-OS have been resolved in various bacteria including the mammalian pathogen *Pseudomonas aeruginosa*. A few core-OS structures of plant-associated *Pseudomonas* strains have been solved to date, but the genetic components of the underlying biosynthesis remained unclear. We conducted a comparative genome analysis of the core-OS gene cluster in *Pseudomonas syringae* pv. *tomato* (*Pst*) DC3000, a widely used model pathogen in plant–microbe interactions, within the *P. syringae* species complex and to other plant-associated *Pseudomonas* strains. Our results suggest a genetic and structural conservation of the inner core-OS but variation in outer core-OS composition within the *P. syringae* species complex. Structural analysis of the core-OS of *Pst* DC3000 shows an uncommonly high phosphorylation and presence of an O-acetylated sugar. Finally, we combined the results of our genomic survey with available structure information to estimate the core-OS composition of other *Pseudomonas* species.

## 1. Introduction

The *Pseudomonas syringae* species complex comprises numerous highly adapted pathovars and is considered an indispensable model for studying plant–bacteria interactions. The genetic diversity of the *P. syringae* complex is reflected by the subdivision into 13 distinct phylogenetic groups [1]. Among them are economically relevant pathogens, which cause substantial yield losses each year [2]. Research aiming to enlighten the mechanism of *P. syringae* pathogenesis contributes to the development of agronomical solutions to prevent and control bacterial disease outbreaks in the field. 

*P. syringae* first colonizes the phylloplane but switches to an endophytic lifestyle to establish an infection. While the prevalence of epiphytic or endophytic growth is strain specific, disease symptoms only emerge when *P. syringae* colonizes the apoplast [1]. During transition between these two lifestyles, bacteria are exposed to profound environmental changes and rely on specific cellular properties to withstand these stresses [3]. The cell wall protects the bacteria from harsh chemical conditions, shields off antimicrobial substances, and contributes to immune evasion processes while simultaneously maintaining cell integrity, nutrient uptake, and material exchange with the environment. In most Gram-negative bacteria, these cell wall characteristics are largely mediated by lipopolysaccharide (LPS), the main component of the outer leaflet of the outer membrane (OM) [4]. The general molecular architecture of LPS is conserved between bacterial species and can be divided into three subdomains: lipid A (LA), core oligosaccharide (core-OS), and O-polysaccharide (OPS) [3]. 

The structural composition of OPS, the most distal LPS domain, is very diverse but often strain-specific and determines the serotypic specificity [5]. Previously analyzed OPS structures of *P. syringae* show a prevalence of l-rhamnose (Rha) residues [6]. Similar to OPS, the outer core-OS is structurally quite variable, while the saccharide composition of the inner core-OS is often conserved and typically contains l-glycero-d-*manno*-heptose and the LPS specific sugar 3-deoxy-d-*manno*-oct-2-ulosonic acid (Kdo) [7]. The LA usually has a di-phosphorylated di-glucosamine backbone which is commonly acylated with up to four primary and from one to three secondary fatty acids. The acylation pattern and length of the acyl chains vary between bacterial families and influence the endotoxic potential of LPS in mammals and humans [8]. For example, the prototypical enterobacterial LA is asymmetrically hexa-acylated with long acyl chains (C12/C14), whereas *Pseudomonas* LA is often penta-acylated with shorter acyl chains (C10/C12) [9,10]. *E. coli* LA is a strong agonist of the human Toll-like receptor 4 (TLR4), while recognition of *P. aeruginosa* LA is weaker due to its shorter acyl chains and is further reduced when penta-acylated [11]. 

In the OM, ionic interactions between negatively charged residues in the LA:core-OS unit and divalent cations result in tight packing of LPS molecules and influence permeability and stability of the OM [12]. They are often targeted by host defenses, e.g., cationic antimicrobial peptides (CAMPs), which disrupt these ionic interactions and destabilize the OM [13]. The core-OS can also be sensed by pattern recognition proteins of the host immune system. Examples in mammals include the membrane proteins brain angiogenesis 51 inhibitor 1 (BAI1), which facilitates phagocytosis of *Enterobacteria* by macrophages [14], and cystic fibrosis transmembrane conductance regulator (CFTR), which binds the outer core-OS of *P. aeruginosa* LPS [15]. The core-OS of *Xanthomonas campestris* pv. *campestris* has been reported to elicit immune responses in *Arabidopsis thaliana* [16] and *Nicotiana tabacum* [17], but no corresponding sensory components have yet been identified in plants. 

The basic core-OS structure can be altered with stoichiometric and nonstoichiometric substitutions to counteract recognition and targeting by host immune components. Such modifications include the addition of phosphoethanolamine to mask negative charges of the phosphates and increase CAMP resistance [18]. The core-OS of *P. aeruginosa* also displays a high degree of nonstoichiometric O-acetylation which is considered characteristic for respiratory and mucosal pathogens [19,20]. O-acetylation might influence cell-surface hydrophobicity and possibly increase resistance to opsonophagocytosis [19,21]. Similarly, the potential influence of O-carbamylation or alanine (Ala) substitutions on immune recognition remains unresolved [10]. 

Biosynthesis of the core-OS takes place at the cytoplasmic face of the inner membrane (IM). The first sugars of the inner core-OS, Kdo, are transferred to the tetra-acylated lipid IV_A_ precursor during the LA biosynthesis. The subsequent addition of further saccharides to the nascent core-OS is mediated by several different membrane-associated glycosyltransferases [8]. In *E. coli*, all enzymes involved in the synthesis of the five known core-OS variations were identified [22]. Similarly, core-OS structure and underlying synthesis genes of *P. aeruginosa* are characterized. Most of the respective genes are located in a proposed gene cluster (genes PA4996-PA5012) and could be associated with a specific function [9,23]. Structural data suggest that two l-glycero-d-*manno*-heptose residues (Hep^I^ and Hep^II^) of the inner core-OS are conserved in all *Pseudomonas* species analyzed to date, with *Pseudomonas cichorii* possibly being the only known exception [24]. Hep^I^ and Hep^II^ are usually highly phosphorylated and substituted with a carbamoyl group which is transferred by the putative carbamoyltransferase *wapO* (PA5005) in *P. aeruginosa* [10,20]. The core phosphates are considered essential for *P. aeruginosa* viability and might contribute to its intrinsic drug resistance [20,25]. The outer core-OS structures vary in their composition between the different *Pseudomonas* species analyzed to date. In *P. syringae*, only the core-OS structure of the pathovar *phaseolicola* was completely analyzed. It contains β-N-acetyl-d-glucosamine (GlcNAc^III^)-(1→2)-α-d-glucose (Glc^I^)-(1→3) and α-l-rhamnose (l-Rha)-(1→6)-β-Glc^II^-(1→4) or α-Kdo^III^-(2→6)-β-Glc^II^-(1→4) chains in the outer core-OS which are linked to the inner core-OS via a →3,4)-α-d-galactosamine (GalN)-(1→3) residue substituted with l-Ala-2) [26]. The relative core-OS sugar content in the *P. syringae* pathovars *maculicola* and *atrofaciens* suggest that the *P. syringae* outer core-OS might be generally defined by the presence of GlcN and Rha residues [26,27,28]. *P. syringae* pv. *tomato* DC3000 (*Pst* DC3000) is a widely used model pathogen for studying molecular microbe–host interactions with *Arabidopsis thaliana*, but yet its core-OS composition and the respective synthesis genes are unknown.

Herein, we elucidate the genetic background of the core-OS synthesis in bacteria from the *P. syringae* species complex and other plant-associated bacteria by comparative analysis of publicly available genomes and predicted proteomes. We identified the core-OS gene cluster in *Pst* DC3000 and could associate most genes with a proposed function. The comparative genome analysis revealed that the gene cluster is highly conserved in *P. syringae* pathovars and predicts a general conservation of the core-OS composition. Supporting this, structural analysis of the core-OS of an OPS-deficient *Pst* DC3000 Δ*wbpL* mutant showed a basically similar composition to the core-OS of *P. syringae* pv. *phaseolicola*. However, in *Pst* DC3000 LPS, we observed a higher degree of core-OS phosphorylation and an O-acetylated sugar. 

## 2. Results

### 2.1. Pst DC3000 Core-OS Gene Cluster Contains an Insertion Sequence Element

In *P. aeruginosa*, the core-OS biosynthetic genes localize in a cluster [20]. Most of the proteins encoded in this gene cluster could be associated with a specific function in core-OS biosynthesis [9]. The position of a putative core-OS gene cluster in the *Pst* DC3000 genome was identified by synteny analysis followed by multiple BLAST searches and pairwise alignments with the corresponding *P. aeruginosa* PAO1 gene products as reference. In total, 15 out of 17 genes in the *P. aeruginosa* core-OS cluster could be matched to sequences between PSPTO_4983 and PSPTO_5003 with predicted protein sequence identities ranging from 86.9% to 55.6% (Table 1). Pairwise alignment of the two unmatched *P. aeruginosa* protein sequences with the sequences of the syntenic *Pst* DC3000 genes PSPTO_4986 (PA4999, *waaL*) or PSPTO_4987 (PA5000, *wapR*) resulted in identities of 19.4% and 7.9%, respectively (Table 1). 

Notably, the putative core-OS cluster of *Pst* DC3000 (PSPTO_4983-PSPTO_5003) includes four additional open reading frames (ORFs, PSPTO_4993-PSPTO_4996). A comparison of the sequences and the gene ontology terms indicates that these might constitute a putative type III effector HopAC1 encoding gene (first segment: PSPTO_4993, second segment PSPTO_4996) which is disrupted by an insertion sequence element (IS-element, ISPsy5 transposase: PSPTO_4994, ISPsy5 ORF: PSPTO_4995). Finally, each gene of the putative core-OS cluster of *Pst* DC3000 (PSPTO_4983-PSPTO_5003) was associated with a putative function by taking gene annotation, gene ontology, and the corresponding function of the *P. aeruginosa* PAO1 orthologs into account (Table 1).

### 2.2. Genes Involved in Synthesis of the Inner Core-OS Are Conserved in Pseudomonas

**Figure 1:**
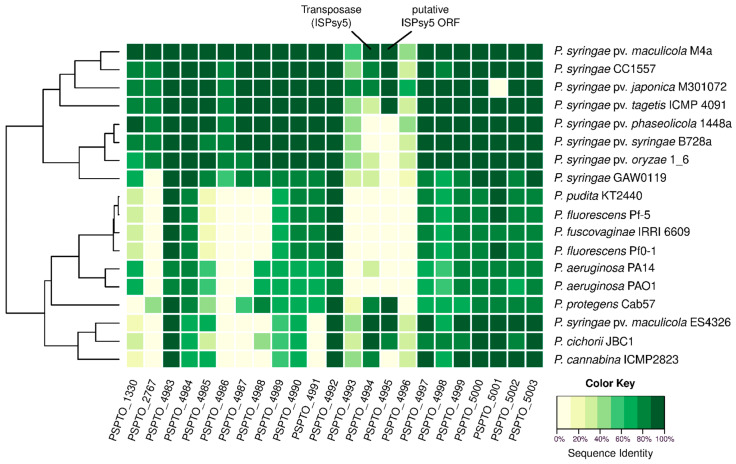
Comparison of core-OS synthesis enzymes. Heatmap of results from an NCBI BLASTP search for homologous core-OS biosynthetic enzymes in different predicted proteomes (Table S1). *Pst* DC3000 sequences were used as reference, e-value cutoff = 10^−9^. Sequence identity values of the BLASTP results are provided in Supplementary Data File 1. Dendrogram according to Euclidean distances calculated from the BLASTP results.

The results of the analysis of the *Pst* DC3000 core-OS gene cluster (PSPTO_4983-PSPTO_5003; Table 1) were used to elucidate core-OS synthesis from bacteria of the *P. syringae* species complex and other representative *Pseudomonas* species. Comparative analysis with predicted proteomes (Table S1) was performed to identify orthologs of known core-OS biosynthesis components and to reveal possible differences. The results suggest a strong conservation of genes associated with inner core-OS compared to outer core-OS synthesis among *Pseudomonas* (Figure 1). 

The respective sequence identities of *P. syringae* pathovars ranged from 100% to 74.5%, whereas the comparison with *P. aeruginosa* PAO1 genes showed the lowest sequence identities (83.5–57.5%) of the analyzed *Pseudomonas* species. Notably, BLASTP search for a PSPTO_5001 protein ortholog in *P. syringae* pv. *japonica* M301072 yielded no hit, while sequence identities for other core-OS synthesis components ranged from 97.4% to 81.0%. Closer inspection of the respective gene sequence shows a potential frame shift resulting in a premature stop codon. Comparison of further core-OS synthesis elements in the *Pst* DC3000 cluster (PSPTO_4983-PSPTO_4992) indicated a conservation of the respective proteins in all analyzed *P. syringae* pathovars (sequence identities: 100.0–60.0%) except *P. syringae* pv. *maculicola* ES4326 (Figure 1). While some of these proteins, e.g., carbamoyltransferase PSPTO_4992 and LA:core-OS transporter PSPTO_4984, seem to be conserved in other *Pseudomonas* species, only single orthologs of the putative glycosyltransferases PSPTO_4991, PSPTO_4988, or PSPTO_4997 could be identified in some predicted *Pseudomonas* proteomes (Figure 1).

**Figure 2:**
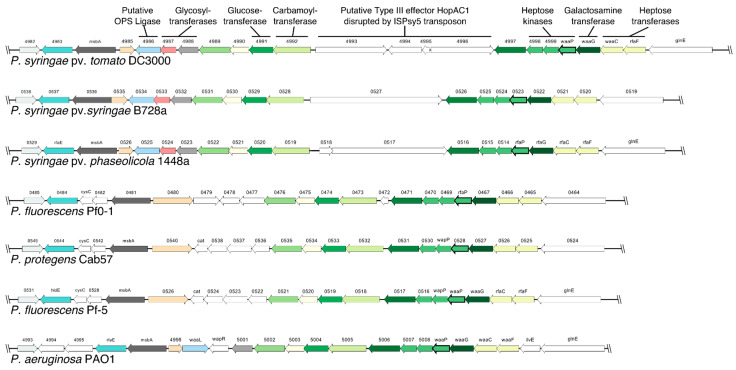
Synteny analysis of the core-OS cluster in *Pseudomonas* strains. Gene structure of the *Pst* DC3000 core-OS cluster and hits from synteny analysis of corresponding genes in selected *Pseudomonas* strains. Matching colors indicate corresponding gene hits.

Three *P. syringae* pathovars and four *Pseudomonas* species were analyzed for synteny within the core-OS gene cluster to identify possible differences in a gene context (Figure 2). The IS-element disrupting the putative type III effector gene *hopAC1* in *Pst* DC3000 is not present in other genomes, but *hopAC1* or homologous sequences were identified in *P. syringae* pv. *syringae* B728a (Psyr_0527) and *P. syringae* pv. *phaseolicola* 1448a (PSPPH_0517/PSPPH_0518). Otherwise, gene synteny is highly conserved among *P. syringae* strains and mainly differs in the sequence and orientation of putative glycosyltransferase and OPS ligase genes upstream of PSPTO_4889. 

In summary, the sequence analysis revealed conserved core-OS gene clusters in *P. syringae* strains and suggests these strains share a common core-OS structure. Available data on the core-OS structure of the *P. syringae* pv. *phaseolicola* and the relative sugar content of pathovar *maculicola* and *atrofaciens* indicate a similar sugar composition [26,27,28]. Next, we analyzed the core-OS structure of the model plant pathogen *Pst* DC3000 in detail.

### 2.3. Structural Analysis of the Pst DC3000 LPS Core-Oligosaccharide

Since we focused on the analysis of the core-OS structure, we isolated LPS of the previously established OPS-deficient *Pst* DC3000 Δ*wbpL* mutant [29], as this yields higher amounts of core-OS. We analyzed the O- and N-deacylated core-LA carbohydrate backbone generated by hydrazinolysis and alkaline hydrolysis (HyKOH-treatment) as well as the core sugar after mild acidic hydrolysis to check for loss of substituents during HyKOH-treatment.

**Figure 3:**
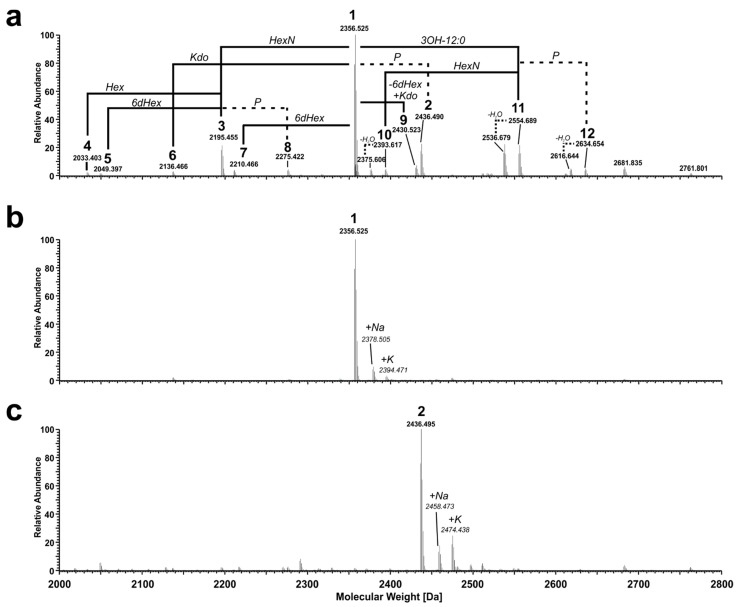
Mass spectrometric analysis of the O- and N-deacylated core-LA carbohydrate backbone of *Pst* DC3000 Δ*wbpL*. (**a**) Molecular species distribution in the mixture of oligosaccharides obtained after hydrazinolysis and alkaline hydrolysis (OS-HyKOH). The charge-deconvoluted spectrum of the MS-analysis performed in negative ion mode is depicted; the observed molecular species are listed in Table 2. MS-spectra of selected pools from the further fractionation by HPAEC containing molecules **1** (**b**) and **2** (**c**), respectively. Molecular masses given in italic style represent sodium (Δm = 21.98 Da) or potassium (Δm = 37.95 Da) adduct ions of the respective base peak.

**Figure 4:**
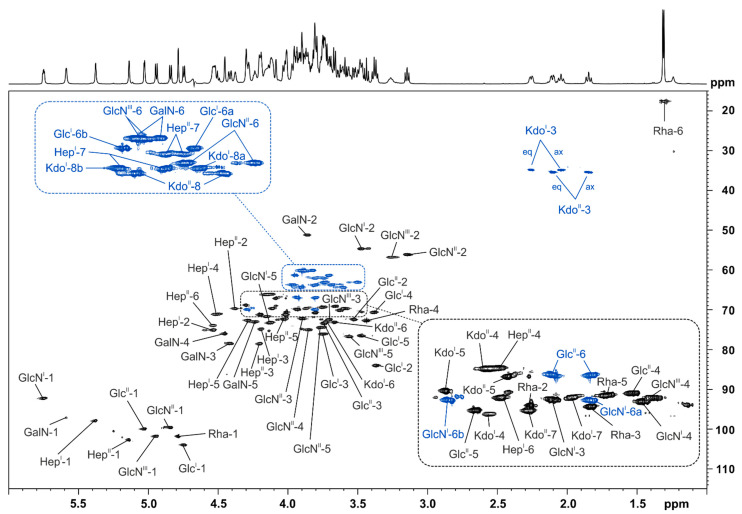
NMR analysis of the major O- and N-deacylated core-LA carbohydrate backbone molecule (**1**) of *Pst* DC3000 Δ*wbpL* after HPAEC-fractionation. Shown above is a section (δ_H_ 6.0–1.0; δ_C_ 115–15) of the ^1^H,^13^C-HSQC NMR spectrum, recorded at 310 K in D_2_O as dept-version (blue: CH_2_-groups, black: CH/CH_3_-groups), including assignment of signals. The corresponding NMR chemical shift data are listed in Table 3, Table 4 and Table 5.

LPS from *Pst* DC3000 Δ*wbpL* was O-deacylated by mild hydrazinolysis and then N-deacylated under strong alkaline conditions. After desalting, the resultant mixture of oligosaccharides (OS-HyKOH; MS spectrum shown in Figure 3a) was further fractionated by HPAEC. A representative analytical HPAEC run of this mixture is depicted in Figure S1. One major (**1**), two minor (**2** and **3**, respectively), and some very minor molecules (**4**–**9**) have been observed; **2** contains one phosphate more than **1**, while **3** lacks one HexN compared to **1**. In addition, not completely deacylated variants of **1**, **2,** and **3** have been found (**10**, **10^anh^**, **11**, **11^anh^**, **12**, and **12^anh^**). All detected species are summarized in Table 2. The MS spectrum of the HPAEC-purified and desalted major observed molecule **1** (pool 2 in HPAEC, Figure S1) is depicted in Figure 3b. Notably, **1** has an exact mass of 2356.525 Da, equivalent to the composition Kdo_2_Hep_2_Hex_2_6dHex_1_HexN_4_P_5_, which is identical to the mass and composition observed for the major core-backbone oligosaccharide in *Pseudomonas syringae* pv. *phaseolicola* [26]. This was further corroborated by one- and two-dimensional NMR experiments on compound **1**. The corresponding ^1^H, ^13^C-HSQC NMR spectrum is shown in Figure 4, and the respective NMR chemical shift data are summarized in Table 3, Table 4 and Table 5. By this, the identical structure of **1** and the major core-backbone oligosaccharide identified in *P. syringae* pv. *phaseolicola* [26] were proven.

**Figure 5:**
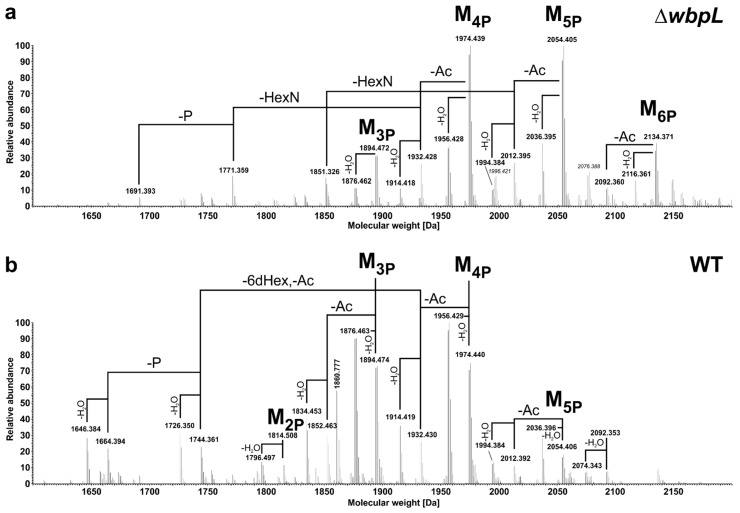
MS analysis of core-OS preparations obtained by hydrolysis with 1% acetic acid from lipopolysaccharide (LPS) of Δ*wbpL* and wild-type *Pst* DC3000. Molecular species distribution in the mixture of core-OS preparations (OS_HOAc_) obtained after treatment of LPS isolated from *Pst* DC3000 Δ*wbpL* (**a**) and wild-type (**b**) with 1% acetic acid. Charge-deconvoluted spectra of the MS-analysis performed in negative ion mode are depicted. Molecular masses given in italic style represent sodium (Δm = 21.98 Da) adduct ions of the respective base peak.

A fraction of molecules with an additional phosphate (**2**; exact mass of 2436.495 Da, Figure 3c) was also isolated by HPAEC (pool 5, Figure S1). Although this pool was represented by multiple peaks in the HPAEC, it was almost homogeneous in the molecular mass. However, the NMR analysis indicated that this pool contains multiple molecules, therefore the positions of the additional phosphate group could not be determined unequivocally, but all of them were monophosphate groups (data not shown). To check for further substituents that are known to be cleaved off during HyKOH-treatment, LPS from *Pst* DC3000 Δ*wbpL* was subjected to hydrolysis with 1% acetic acid. This treatment cleaves the linkage between LA and core-OS under elimination of one Kdo (Kdo^II^) but leaves N-alanyl-, N-/O-acetyl-, and O-carbamoyl-residues as well as diphosphate bonds intact [30]. The MS spectrum of the desalted core-OS preparation (OS_HOAc_) is depicted in Figure 5a. Compared to the core-OS molecules observed in *P. syringae* pv. *phaseolicola* [26], two major differences are obvious: the core-OS of *Pst* DC3000 Δ*wbpL* bears more phosphate moieties (up to six instead of up to four in *P. syringae* pv. *phaseolicola*) and contains additional acetyl moieties.

The structure of the basic core-OS molecule (M) has the following composition as judged by calculated masses: Kdo_1_Hep_1_HepCm_1_Hex_2_6dHex_1_HexN_2_Ala_1_Ac_3_ with varying numbers of phosphate residues (three to six; M_3P_ to M_6P_, respectively). Calculated and observed masses for core-OS molecules present in this preparation are summarized in Table 6. To prove that the additional two predominant acetylations are not an effect of the *wbpL*-knockout and a resulting loss of OPS, the same treatment and analysis was performed with LPS isolated from wild-type *Pst* DC3000. The MS spectrum of this desalted core-OS preparation is depicted in Figure 5b and major core-OS molecules present are summarized in Table 6. Molecules with a mass difference of −18 Da are the result of the known release of water from the reducing Kdo under such chemical treatment conditions.

This analysis verified that these acetylations also occur in core-OS of WT *Pst* DC3000. The majority of observed molecules was similar in preparations of both strains (Table 6), albeit molecular species were observed in significantly different relative abundances. One major difference was the higher content of phosphorylation in OS_HOAc_ obtained from *Pst* DC3000 Δ*wbpL* LPS. In the OS_HOAc_ of this mutant, the major molecules were M_4P_ and M_5P_, as well as their variants with one acetyl group less (-Ac) and respective anhydro-compounds (-H_2_O). This is shifted in WT to molecules of M_3P_ and M_4P_ type, respectively. Furthermore, only in the preparation of the Δ*wbpL* mutant molecular species lacking one hexosamine (-HexN) are significantly present (1851.326 Da, 1771.359 Da; Figure 5a). By contrast, OS_HOAc_ obtained from *Pst* DC3000 WT LPS contained molecular species to a significant degree, in which the 6-deoxyhexose together with one acetyl moiety is lacking (-6dHex, -Ac; 1744.361 Da, 1664.394 Da, and their respective anhydro-variants; Figure 5b). Notably, in these 6dHex (Rha)-lacking molecules only one acetyl moiety is present, pointing to the potential presence of these modifications at this terminal residue. 

**Figure 6:**
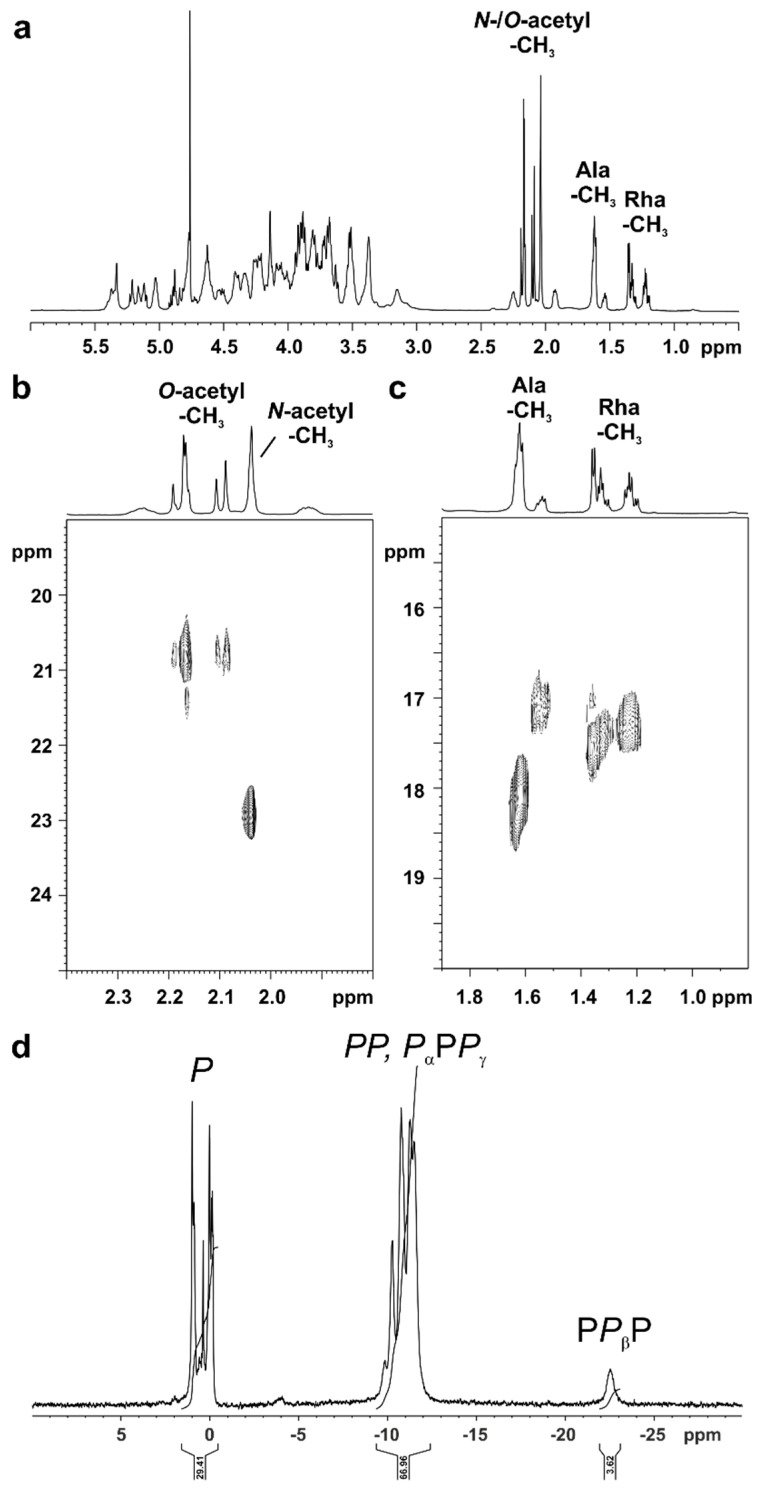
NMR analysis of the OS_HOAc_ preparation of *Pst* DC3000 Δ*wbpL* LPS. (**a**) Shown above is the full ^1^H NMR spectrum (δ_H_ 6.0–0.5; recorded in D_2_O at 300 K); regions for signals resulting from N-/O-acetyl groups, Ala-CH_3_, and Rha-CH_3_, respectively, are indicated. (**b**,**c**) Sections of the ^1^H,^13^C-HSQC NMR spectrum showing regions with ^1^H,^13^C-cross correlations for N-/O-acetyl groups (δ_H_ 2.40–1.80; δ_C_ 25–19; (**b**)) as well as Ala-CH_3_ and Rha-CH_3_ moieties (δ_H_ 1.90–0.80; δ_C_ 20–15; (**c**)) are shown. The signal assignment is discussed in the text. (**d**) Shown above is the ^31^P NMR spectrum (δ_P_ 10-(-30)) recorded in D_2_O at 300 K. Monophosphates (*P*) are represented by the group of signals between δ_P_ 2 and −1 ppm, diphosphates (*PP*) as well as *P*_α_ and *P*_γ_ of triphosphates appear between δ_P_ −9 and −12 ppm, and *P*_β_ of triphosphates is represented by the broad signal between δ_P_ −22 and −23 ppm.

Despite the known complexity of such core-OS preparations due to the high degree of structural heterogeneity (e.g., caused by two outer core glycoforms (w/wo HexNAc), varying degree of phosphorylation, and anhydro versions of all molecules) we analyzed the OS_HOAc_ preparation derived from *Pst* DC3000 Δ*wbpL* by NMR, especially aiming to identify the position of the additional acetyl substituents. The full ^1^H NMR spectrum is shown in Figure 6a, and the region for CH_3_ groups of the ^1^H, ^13^C-HSQC NMR is displayed in Figure 6b,c. Besides the presence of an N-acetyl group (δ_H_ 2.04; δ_C_ 23.0) multiple O-acetyl groups (δ_H_ 2.20–2.08; δ_C_ 20.9/20.8) can be detected (see Figure 6b). The major portion of the N-alanyl (Ala) CH_3_ group is represented by two overlapping doublets at δ_H_ 1.63/1.62 with δ_C_ 18.2 (most likely in the major occurring glycoform including the terminal HexNAc). A minor portion can be detected again as two overlapping doublets at δ_H_ 1.55/1.54 with δ_C_ 17.2 (most likely derived from the minor glycoform lacking the terminal HexNAc).

**Figure 7:**
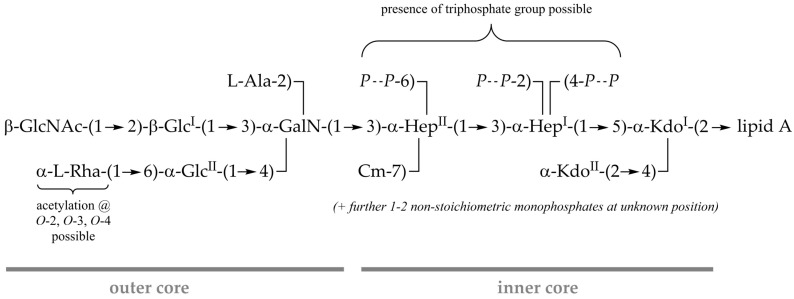
Scheme of the core-OS of *Pst* DC3000 LPS. It has the same basic structure as the core-OS identified in *P. syringae* pv. *phaseolicola* (glycoform 1) [26], but for *Pst* DC3000 core-OS the observed degree of phosphorylation is significantly higher. Position 2 and 4 of Hep^I^ or position 6 of Hep^II^ are occupied by a diphosphate group in a significant proportion (dashed lines indicate nonstoichiometric substitution) and further nonstoichiometric monophosphates at so far unknown positions can be present. To a lesser extent, triphosphate groups are present as well. The terminal Rha moiety is O-acetylated with up to two acetyl groups.

Interestingly, multiple doublets between δ_H_ 1.38–1.18, all with corresponding carbons at δ_C_ 17.6–17.2, point to the presence of various versions of the terminal Rha residue (Figure 6c). Furthermore, the presence of an O-carbamoyl group was indicated in the ^13^C-NMR spectrum of this OS_HOAc_ preparation (not shown) at δ_C_ 158.9 (compared to δ_C_ 159.4 in the core of *Pseudomonas syringae* pv. *phaseolicola* [26]). Its analysis by ^31^P NMR revealed a significant proportion of di- and to a minor extent even triphosphates (appr. 1:1:0.1 as judged by sum-integration of signals). Diphosphates and *P*_α_ and *P*_γ_ of triphosphates are represented by the group of signals between δ_P_ −9 and −12 ppm, *P*_β_ of triphosphates by the broad signal between δ_P_ −22 and −23 ppm (Figure 6d). Unfortunately, neither attempt aiming for the isolation of homogeneous core-OS molecules by HPAEC directly from this preparation nor after dephosphorylation by HF treatment (which also partially cleaves off O-acetyl residues) has been successful (data not shown). Therefore, the final assignment of the positions of these diphosphates and the combinations of O-acetylation of the Rha moiety remains partially elusive. The chemical structure of the core-OS of *Pst* DC3000 LPS as revealed here is summarized in Figure 7. 

## 3. Discussion

In this study, we identified the core-OS gene cluster in *Pst* DC3000 and analyzed publicly available sequence data to compare the genetic background of core-OS synthesis in the *P. syringae* species complex and other plant-associated *Pseudomonas* bacteria. The core-OS cluster is generally conserved within *Pseudomonas*. While the gene content is very similar in most *P. syringae* pathovars, variations in genes responsible for outer core-OS synthesis are apparent in other bacteria of the species complex. Previous core-OS structural analyses of *P. syringae* suggest that GalN and Rha residues are characteristic for the outer core region [26,27,28]. According to our analysis, GalN might be present in all *Pseudomonas* core-OS. The putative core-OS Rha transferase of *Pst* DC3000 (PSPTO_1330) is only conserved among *P. syringae* pathovars but not in the *P. syringae* species complex [26,27,28]. While other Rha transferases might be involved in core-OS synthesis in these strains, it is likely that they lack a Rha residue as observed in other *Pseudomonas* species, e.g., *P. tolaasii* [31]. 

The presence of two putative Hep transferases (PSPTO_5002, PSPTO_5003), three Hep kinases (PSPTO_4998-PSPTO_5000), a putative GalN transferase (PSPTO_5001), and a putative carbamoyltransferase in all *Pseudomonas* genomes suggests that they possess a common proximal core-OS structure with →3)-α-GalN-(1→3)-α-Hep^II^-(1→3)-α-Hep^I^-(1. Both Hep residues are phosphorylated and a carbamoyl residue is linked to Hep^II^. The inner core-OS phosphates are essential for the viability and drug resistance of *P. aeruginosa* [25,32]. The conservation of the high degree of phosphorylations might be one of the factors responsible for the intrinsic resistance against harsh environmental conditions and the resulting versatility of bacteria belonging to the genus *Pseudomonas* [33]. Notably, although structural analysis of the *P. cichorii* 5707 core-OS indicated that it lacks Hep residues and phosphates [24], the corresponding biosynthetic genes are conserved in the *P. cichorii* JBC1 genome and are presumably functional. Possibly, this is due to genetic differences between *P. cichorii* strains. Alternatively, since the core-OS analysis of *P. cichorii* 5707 was conducted with bacteria cultivated in minimal media [34], the adaptation of bacteria to such conditions could result in a loss of Hep residues in the core-OS. 

In accordance with the results from the genomic survey, analysis of the *Pst* DC3000 core-OS structure revealed that it is mostly identical to the *P. syringae* pv. *phaseolicola* core-OS analyzed by Zdorovenko et al. [26]. The major observed molecule after HyKOH-treatment was of same composition as the glycoform 1 observed in this *P. syringae* pathovar. Glycoform 2 observed in the *P. syringae* pv. *phaseolicola* rough-type strain (GSPB 711) used in that study, in which the terminal Rha is exchanged to a Kdo moiety, was only marginally observed in *Pst* DC3000 (molecule **9**). Moreover, a part of molecules (molecules **3** and **8**; and to a much lesser extent, molecules **4** and **5**) lacked the terminal β-GlcNAc residue. However, this might be caused by the *wbpL*-knockout, which impairs OPS addition [29], since comparable variants of the core-OS after mild acidic hydrolysis are only observable for the OS_HOAc_ of the *wbpL*-knockout strain. In the analogous preparation from LPS of the isogenic wild-type strain, this substituent is stoichiometrically present. Here, in turn, to some extent the terminal, O-acetylated Rha residue is lacking. The core-OS of some *P. aeruginosa* strains [20,23] and of *P. fluorescens* ATCC 49271 [35] have been described to display a high degree of nonstoichiometric O-acetylation. The specific positions of these O-acetylations in *P. aeruginosa* strains could not be completely assigned, but at least one was located at O-2 of a terminal Rha residue [30], another at O-6 of the Glc^II^ [20]. A random distribution of O-acetylation at O-2, O-3, and O-4 of the terminal Rha has been observed in other studies of *P. aeruginosa* core-OS structures [36,37]. For the core-OS of the *Pst* DC3000 LPS, the assignment of O-acetyl groups to specific positions was also not completely possible. However, our data clearly suggest the presence of O-acetyl groups at the terminal Rha and a mixture of mono- and di-O-acetylated (2,3-di-O-acetyl, 2,4-di-O-acetyl, 3,4-di-O-acetyl) molecules. O-acetylation is associated with specialized respiratory and mucosal pathogens and might influence cell surface hydrophobicity and possibly increase resistance to opsonophagocytosis [19,21,30]. However, the investigation of its influence on plant-host colonization requires identification of the respective O-acetyl transferases, their specific knockout, and a subsequent comparative analysis of mutant and wild-type bacteria in in vivo assays. Moreover, *P. aeruginosa* is known to contain a high phosphorus content in its LPS core-OS, especially the inner core-OS, present as mono-, di-, and even tri-phosphates at multiple positions [30,38]. By contrast, the core-OS of *P. syringae* pv. *phaseolicola* contains only three stoichiometrically defined phosphates (position 2 and 4 of Hep^I^, position 6 of Hep^II^), whereas the phosphate at position 2 of Hep^I^ can be in part substituted with a phosphoethanolamine as well. Our analysis of the *Pst* DC3000 core-OS showed that here the phosphorylation pattern is more similar to that of *P. aeruginosa*. A high degree of diphosphate groups and the presence of triphosphates was observed. However, attempts to determine the exact sites of attachment of these groups by NMR analysis failed due to the high degree of heterogeneity of core-OS molecules, which is caused by the occurrence of Kdo in multiple forms and, most likely, by nonstoichiometric phosphorylation resulting in the splitting of the signals. In light of the same basic structure of the core-OS for *Pst* DC3000 as elucidated in this work in comparison with the core-OS found in *P. syringae* pv. *phaseolicola*, the disruption of the putative *hopAC1* homolog by an IS-element, which was also described in previous reports of type III effector proteins in *Pst* DC3000 [39], is unlikely to have a major influence on core-OS biosynthesis. Given the critical role of the core-OS for bacterial viability and potentially virulence in interactions with host plants it will be interesting to see in future studies whether the structural features observed here for the *Pst* DC3000 LPS core-OS are common to other plant-adapted *Pseudomonas* species.

## 4. Materials and Methods

### 4.1. Bacterial Strains and Growth Conditions

*Pst* DC3000 WT or Δ*wbpL* were grown at 26 °C in King’s B (KB) media liquid culture with shaking (230 rpm) or on KB agar plates with 2% (*w*/*v*) agar.

### 4.2. Hot Phenol-Water and Phenol-Chloroform-Petroleum Ether Extraction of LPS

Preparation of LPS from *Pst* DC3000 WT and Δ*wbpL* is described in or was performed according to reference [29]. 

### 4.3. Alkaline Degradation of the Lipopolysaccharide

LPS (151.8 mg) of *Pst* DC3000 Δ*wbpL* was treated with anhydrous hydrazine (1.5 mL) in a 10 mL rim rolled bottle (Macherey-Nagel, Düren, Germany) for 2 h at 37 °C. Afterwards, approximately 5 mL ice-cold acetone were added dropwise and precipitation was allowed overnight at −50 °C. The complete material was transferred into a 30 mL Nalgene™ Oak Ridge high-speed centrifuge tube (FEP; Thermo Scientific Nalgene Products, Rochester, NY, United States), filled with ice-cold acetone, and centrifuged (47,000× *g*) for 30 min at 4 °C. The precipitate was washed four times with ice-cold acetone, all supernatants were discarded. The sediment was dried under a nitrogen stream, transferred with Millipore-grade water (MP-water) into a 10-mL rim-rolled bottle and lyophilized (yield: 114.4 mg). Next, 2.28 mL 4 M KOH were added, and the sediment suspended using ultrasonics. The solution was purged for 15 min with a slight stream of nitrogen at RT and then heated for 18 h at 120 °C. After cooling to RT, the solution was transferred into a high strength centrifuge tube (chemically strengthened borosilicate glass, Type 1, Class B, Kimble-Chase, Rockwood, TN, United States) and centrifuged (2500× *g*) for 15 min at 4 °C. The pH of the resulting supernatant was adjusted with 4 M HCl to approximately 6.0 and afterwards extracted three times with 5 mL dichloromethane each (centrifugation: (1) 2500 *g*, for 15 min at 4 °C; (2) 2500 *g*, for 30 min at 4 °C; and (3) 5000 *g*, for 30 min at 4 °C). The aqueous phase was evaporated under reduced pressure and lyophilized. Further purification was achieved by gel-permeation chromatography on a Sephadex G-50 column (2.5 × 50 cm; GE Healthcare Bio-Sciences, Uppsala, Sweden) using pyridine–acetic acid–water (8:20:2000 (*vol*/*vol*), pH~4.7) as eluent. Oligosaccharides were monitored by a Knauer differential refractometer. For application to the column, the material was resuspended in 2 mL MP-water and centrifuged (371× *g*) for 5 min at 20 °C. Fractions containing the O- and N-deacylated core-LA carbohydrate backbone molecules (OS-HyKOH) were collected and lyophilized (yield: 57 mg).

A portion of the isolated OS-HyKOH mixture (dissolved 20 mg/mL in MP-water; approximately 10 mg per run) was fractionated by high-performance anion-exchange chromatography (HPAEC) on a semipreparative CarboPac PA1 column (250 × 9 mm; Dionex, Sunnyvale, CA, USA) using the following gradient (mobile phase A: MP-water; mobile phase B: 1 M NaOAc pH 6.0): 1% B for 5 min, linear gradient raising from 1 to 15% B (5–20 min), maintaining at 15% B for 10 min, linear gradient raising from 15 to 38% B (30–50 min), followed by a linear gradient raising from 38 to 100% B (50–90 min), and held at 100% B for further 30 min. Afterwards, the column was run for 10 min at the initial condition (1% B) to prepare for next injection. The flow rate was 2 mL/min and 2 mL fractions were collected. Selected fractions were analyzed by HPAEC using pulsed amperometric detection with postcolumn addition of 0.5 M NaOH (Dionex) on an analytical CarboPac PA1 column (250 × 4.0 mm) using the same eluents with a flow rate of 1 mL/min with the following gradient: 1% B for 5 min, linear gradient raising from 1 to 15% B (5–20 min), maintaining at 15% B for 15 min, linear gradient raising from 15 to 38% B (35–55 min), followed by a linear gradient raising from 38 to 100% B (55–70 min), and held at 100% B for 8 min more. Afterwards, the column was run for 8 min at initial condition (1% B) to prepare for the next injection. Appropriate fractions were combined and lyophilized. Desalting was performed on a Sephadex G-50 column as described above. Yields resulting from separation of 27.8 mg OS-HyKOH mixture in three runs were as follows: pool 1, 0.78 mg; pool 2, 8.81 mg; pool 3, 1.28 mg; pool 4, 2.62 mg; pool 5, 3.23 mg; and pool 6, 1.27 mg. For assignment of molecules to HPAEC pools, see Table 2 and Figure S1.

### 4.4. Mild-Acid Degradation of the Lipopolysaccharide

LPS (26 mg for *Pst* DC3000 WT; 2 × 55 mg for *Pst* DC3000 Δ*wbpL*) was dissolved in aqueous 1% HOAc (3 mg/mL) and heated for 1.5 h at 100 °C. The further procedure is described for core-OS from *Pst* DC3000 Δ*wbpL*: to enable parallel extraction of LA (not further discussed here, for composition see reference [40]), samples were equally portioned in four 30 mL Nalgene™ Oak Ridge high-speed centrifuge tubes (FEP; Thermo Scientific Nalgene Products, Rochester, NY, United States), and chloroform/methanol 8:2 (*v*/*v*) was added until the tubes were completely filled and thoroughly mixed. After centrifugation (6000× *g*) for 10 min at 4 °C, the lower organic phase was collected. The tubes were refilled with chloroform, thoroughly mixed, and centrifuged again. This procedure was repeated four times in total. The organic phase from the initial chloroform-/methanol-extraction and the first three chloroform extractions were sequentially combined in a pear-shaped flask and reduced to residual water by evaporation under reduced pressure. The chloroform of the last extraction was used to solubilize the material in the pear-shaped flask again (with ultrasonic) and equally portioned into four 30 mL Nalgene™ tubes. Remaining material in the pear-shaped flask was transferred with 4 mL chloroform/methanol 8:2 (*v*/*v*) in total into these tubes as well, using ultrasonic for solubilization. These combined organic phases (containing LA) were washed three times with water and finally evaporated under reduced pressure. All aqueous phases (containing core OS) were combined, neutralized with 1 M NaOH (in Δ*wbpL* core-OS preparation), evaporated under reduced pressure to remove residual organic solvents, and finally lyophilized. The core-OS preparation was further purified by gel permeation chromatography (GPC) on Sephadex G-50 (GE Healthcare Bio-Sciences, Uppsala, Sweden) on a column (2.5 × 50 cm) as described [41]. This yielded 4.5 mg core-OS of *Pst* DC3000 WT and 45.8 mg of *Pst* DC3000 Δ*wbpL*, respectively.

### 4.5. NMR Spectroscopy

Deuterated solvents were purchased from Deutero GmbH (Kastellaun, Germany). NMR spectroscopic measurements were performed in D_2_O at stated temperatures on a Bruker Avance^III^ 700 MHz (equipped with an inverse 5 mm quadruple-resonance Z-grad cryoprobe). Acetone was used as an external standard for calibration of ^1^H (δ_H_ = 2.225) and ^13^C (δ_C_ = 30.89) NMR spectra [42], and 85% of phosphoric acid was used as an external standard for calibration of ^31^P NMR spectra (δ_P_ = 0.00). All data were acquired and processed by using Bruker TOPSPIN V 3.1 or higher (Bruker BioSpin Corporation, Billerica, MA, USA). ^1^H NMR assignments were confirmed by 2D ^1^H,^1^H-COSY, and total correlation spectroscopy (TOCSY) experiments. ^13^C NMR assignments were indicated by 2D ^1^H,^13^C-HSQC, based on the ^1^H NMR assignments. Inter-residue connectivity and further evidence for ^13^C assignment were obtained from 2D ^1^H,^13^C-heteronuclear multiple bond correlation and ^1^H,^13^C-HSQC-TOCSY. Connectivity of phosphate groups were assigned by 2D ^1^H,^31^P-HMQC and ^1^H,^31^P-HMQC-TOCSY.

### 4.6. Mass Spectrometry

All samples were measured on a Q Exactive Plus mass spectrometer (Thermo Fisher Scientific, Bremen, Germany) using a Triversa Nanomate (Advion, Ithaca, NY, USA) as ion source. All measurements were performed in negative-ion mode using a spray voltage of −1.1 kV. Samples were dissolved in a water/propan-2-ol/trimethylamine/acetic acid mixture (50:50:0.06:0.02, *v*/*v*/*v*/*v*) in a final concentration of approximately 0.07 mg/mL (mixtures) or 0.03 mg/mL (HPAEC pools). The mass spectrometer was externally calibrated with glycolipids of known structure. All mass spectra were charge deconvoluted and given mass values that refer to the monoisotopic mass of the neutral molecules. Deconvoluted spectra were computed using Xtract module of Xcalibur 3.1. software (Thermo Fisher Scientific, Bremen, Germany).

### 4.7. Sequence Analysis

Reciprocal BLAST experiments of protein-coding regions in the *Pst* DC3000 core-OS cluster (UniProt proteome ID: UP000002515) were conducted against the predicted proteomes listed in Table S1. A Python script was used to identify homologs of the query sequences (NCBI BLASTP) in each proteome which were set up as individual local databases. The sequence of the first hit was retrieved by the algorithm and the sequence identity was calculated from the quotient of hit sequence length and corresponding identities. Heatmaps were generated from the sequence identity values of the BLASTP results (Supplementary Data File 1), and dendrograms were calculated from the corresponding Euclidean distances. All scripts are available online (https://gitlab.com/alexander.kutschera/quickblast, accessed on 11 January 2021).

Gene synteny was analyzed using SyntTax with standard settings [43]. The graphical output of multiple analyses was merged and modified with Inkscape 0.92.

## Figures and Tables

**Table 1:** Genes of the core oligosaccharide (core-OS) cluster and putative function of the encoded enzymes in *Pseudomonas syringae* pv. *tomato* DC3000 in comparison to *P. aeruginosa* PAO1 based on publicly available data.

Identifier	Annotation ^1^	Putative function in *Pst* DC3000	PAO1 Equivalent	Putative/Proven Function in PAO1	Identity %
**Genes within core-OS cluster**					
PSPTO_4983	Lipopolysaccharide biosynthesis protein RfaE	Heptose biosynthesis	*hldE* (PA4996)	Heptose biosynthesis	86.9
PSPTO_4984	Lipid A ABC transporter, ATP-binding/permease protein	Lipid-A:core-OS transport	*msbA* (PA4997)	Lipid-A:core-OS transport	83.8
PSPTO_4985	Toluene tolerance protein	Unknown	PA4998	Kinase	55.6
PSPTO_4986	Membrane protein	Putative OPS ligase	*waaL* (PA4999) ^2^	OPS ligase	No hit/19.4 ^3^
PSPTO_4987	Hypothetical protein	WbcX-like glycosyltransferase	*wapR* (PA5000) ^2^	Glycosyltransferase (Rha)	No hit/7.9 ^3^
PSPTO_4988	Hypothetical protein	RfaB family glycosyltransferase	PA5001	Glycosyltransferase	73.6
PSPTO_4989	Hypothetical protein	PIG-L family deacetylase	PA5002	Unknown	65.5
PSPTO_4990	Hypothetical protein	GNAT family N-acetyltransferase	PA5003	Unknown	69.1
PSPTO_4991	Glycoside hydrolase family protein	Glycosyltransferase (Glc^II^)	*wapH* (PA5004)	Glycosyltransferase (Glc^II^)	71.2
PSPTO_4992	Carbamoyltransferase family protein	Cm-(→7) carbamoyltransferase	*wapO* (PA5005)	Carbamoyltransferase	86.5
PSPTO_4993	Hypothetical protein	Type III effector HopAC1 (segment)			No hit
PSPTO_4994	ISPsy5, transposase	ISPsy5, transposase			No hit
PSPTO_4995	ISPsy5, Orf1	ISPsy5, Orf1			No hit
PSPTO_4996	Hypothetical protein	Type III effector HopAC1 (segment)			No hit
PSPTO_4997	Hypothetical protein	Unknown	PA5006	Kinase	67.7
PSPTO_4998	Lipopolysaccharide biosynthesis protein	Heptose kinase	*wapQ* (PA5007)	Heptose kinase	57.5
PSPTO_4999	Lipopolysaccharide core biosynthesis protein	Heptose kinase	*wapP* (PA5008)	Heptose kinase	76.2
PSPTO_5000	Lipopolysaccharide core biosynthesis protein WaaP	Heptose kinase	*waaP* (PA5009)	Heptose kinase	79.5
PSPTO_5001	Lipopolysaccharide core biosynthesis protein WaaG	α-GalN-(1→3) Glycosyltransferase	*wapG* (PA5010)	Glycosyltransferase (GalN)	78.6
PSPTO_5002	Lipopolysaccharide heptosyltransferase	α-Hep^I^-(1→5) Glycosyltransferase	*waaC* (PA5011)	Glycosyltransferase (Hep^I^)	74.9
PSPTO_5003	ADP-heptose--LPS heptosyltransferase II	α-Hep^II^-(1→3) Glycosyltransferase	*waaF* (PA5012)	Glycosyltransferase (Hep^II^)	83.5
**Genes outside of the cluster**					
PSPTO_1330	Glycosyltransferase family protein	Glycosyltransferase α-l-Rha-(1→6)	*migA* (PA0705)	Glycosyltransferase (Rha1→6)	63.9
PSPTO_2767	Lipopolysaccharide core biosynthesis domain protein				No hit

^1^ according to www.pseudomonas.com accessed on 11.01.2021; ^2^ located outside of the cluster; ^3^ BLAST yielded no hit, identity result from pairwise alignment of syntenic gene sequences.

**Table 2:** Mass spectrometric analysis of the O- and N-deacylated core-LA carbohydrate backbone of *Pst* DC3000 Δ*wbpL*. Summary of calculated monoisotopic neutral masses and observed molecular masses [Da] in the OS-HyKOH preparation, the corresponding MS spectrum is depicted in Figure 3a. Accuracy of the measurement is stated as Δppm; anh = anhydro.

Molecule	Composition	Calculated Mass [Da]	Observed Mass [Da]	Error [ppm]	HPAEC *
**4**	Kdo_2_Hep_2_Hex_1_6dHex_1_HexN_3_P_5_	2033.403	2033.403	0.0	pool 4
**5**	Kdo_2_Hep_2_Hex_2_HexN_3_P_5_	2049.398	2049.397	−0.5	pool 3
**6**	Kdo_1_Hep_2_Hex_2_6dHex_1_HexN_4_P_5_	2136.466	2136.466	0.0	pool 1
**3**	Kdo_2_Hep_2_Hex_2_6dHex_1_HexN_3_P_5_	2195.456	2195.455	−0.5	pool 4
**7**	Kdo_2_Hep_2_Hex_2_HexN_4_P_5_	2210.467	2210.466	−0.5	pool 3
**8**	Kdo_2_Hep_2_Hex_2_6dHex_1_HexN_3_P_6_	2275.422	2275.422	0.0	pool 6
**1**	Kdo_2_Hep_2_Hex_2_6dHex_1_HexN_4_P_5_	2356.524	2356.525	0.4	pool 2/3
**10^anh^**	Kdo_2_Hep_2_Hex_2_6dHex_1_HexN_3_P_5_[12:0(3-OH)]–*H_2_O*	2375.607	2375.606	−0.4	**
**10**	Kdo_2_Hep_2_Hex_2_6dHex_1_HexN_3_P_5_[12:0(3-OH)]	2393.618	2393.617	−0.4	**
**9**	Kdo_3_Hep_2_Hex_2_HexN_4_P_5_	2430.525	2430.523	−0.8	pool 4
**2**	Kdo_2_Hep_2_Hex_2_6dHex_1_HexN_4_P_6_	2436.491	2436.490	−0.4	pool 5
**11^anh^**	Kdo_2_Hep_2_Hex_2_6dHex_1_HexN_4_P_5_[12:0(3-OH)]*–H_2_O*	2536.676	2536.679	1.2	**
**11**	Kdo_2_Hep_2_Hex_2_6dHex_1_HexN_4_P_5_[12:0(3-OH)]	2554.686	2554.689	1.2	**
**12^anh^**	Kdo_2_Hep_2_Hex_2_6dHex_1_HexN_4_P_6_[12:0(3-OH)]*–H_2_O*	2616.642	2616.644	0.8	**
**12**	Kdo_2_Hep_2_Hex_2_6dHex_1_HexN_4_P_6_[12:0(3-OH)]	2634.653	2634.654	0.4	**

* see Figure S1, ** these monoacylated molecules elute at later retention times, and pools were of minor yield.

**Table 3:** ^1^H NMR chemical shift data of **1** recorded in D_2_O at 310 K.

**Residue**	**H1**	**H2**	**H3**	**H4**	**H5**	**H6_a_**	**H6_b_**	**H7_a_**	**H7_b_**
→6)-α-GlcN^I^-(1→*P*	5.77–5.73	3.50–3.45	3.96–3.91	3.65–3.60	4.15–4.10	3.83–3.78	4.31–4.27		
→6)-β-GlcN^II^4*P*-(1→	4.84 [d, 8.5 Hz]	3.17–3.12	3.92–3.87	3.88–3.83	3.79–3.73	3.52–3.48	3.76-3.71		
→3)-α-Hep^I^2*P*4*P*-(1→	5.39–5.36	4.54–4.51	4.22–4.17	4.54–4.48	4.30–4.26	4.14–4.09		3.83–3.79	3.99–3.94
→3)-α-Hep^II^6*P*-(1→	5.15–5.12	4.39–4.36	4.21–4.18	4.13–4.09	4.05–4.00	4.56–4.50		3.77–3.71	3.82–3.77
→3,4)-α-GalN-(1→	5.60–5.57	3.88–3.83	4.43–4.39	4.46–4.44	4.24–4.21	3.84–3.80	3.93–3.88		
→2)-β-Glc^I^-(1→	4.75 [d, 7.9 Hz]	3.38–3.34	3.77–3.73	3.40–3.36	3.50–3.45	3.73–3.69	3.97–3.93		
→6)-α-Glc^II^-(1→	5.03 [d, 7.9 Hz]	3.55–3.50	3.76–3.72	3.68–3.63	4.22–4.18	3.81–3.78	3.95–3.92		
β-GlcN^III^-(1→	4.94 [d, 8.4 Hz]	3.29–3.22	3.74–3.68	3.61–3.55	3.58–3.53	3.95–3.85	3.95–3.85		
α-l-Rha-(1→	4.80–4.77	4.03–4.00	3.81–3.79	3.46–3.42	3.77–3.72	1.31 [d, 6.1 Hz]			
**Residue**	**H3_eq_**	**H3_ax_**	**H4**	**H5**	**H6**	**H7**	**H8_a_**	**H8_b_**	
→4,5)-α-Kdo^I^-(2→	2.30–2.23	2.04 [dd, 12.3, 12.0 Hz]	4.17–4.12	4.31–4.27	3.74–3.71	3.89–3.85	3.63–3.58	3.93–3.88	
α-Kdo^II^-(2→	2.11 [dd, 12.9, 4.3 Hz]	1.85 [dd, 12.9, 12.6 Hz]	4.18–4.13	4.09–4.07	3.68–3.65	4.04–3.99	3.71–3.66	3.99–3.94	

**Table 4:** ^13^C NMR chemical shift data of **1** recorded in D_2_O at 310 K.

Residue	C1	C2	C3	C4	C5	C6	C7	C8
→6)-α-GlcN^I^-(1→*P*	92.4–92.2	54.6	69.9	70.1	73.3	69.9		
→6)-β-GlcN^II^4*P*-(1→	99.6	56.1	72.1	75.0	74.4	63.1		
→4,5)-α-Kdo^I^-(2→	n.d.	n.d.	34.8	71.6	68.8	72.9	69.7	64.3
α-Kdo^II^-(2→	n.d.	n.d.	35.5	66.1	67.1	73.1	71.2	63.7
→3)-α-Hep^I^2*P*4*P*-(1→	98.0	75.0	74.8	71.1	72.6	69.7	63.7	
→3)-α-Hep^II^6*P*-(1→	102.8	69.7	78.3	66.1	72.3	74.0	62.0	
→3,4)-α-GalN-(1→	97.2	51.1	78.4	75.9	73.0	60.1		
→2)-β-Glc^I^-(1→	104.0	84.0	76.0	70.6	76.4	61.3		
→6)-α-Glc^II^-(1→	100.0	72.3	73.3	69.1	71.1	66.9		
β-GlcN^III^-(1→	101.8	56.7	72.4	69.6	76.6	60.2		
α-l-Rha-(1→	101.9	70.6	70.8	72.7	69.4	17.7		

**Table 5:** ^31^P NMR chemical shift data of **1** recorded in D_2_O at 310 K.

Residue	^31^P Chemical Shift [ppm]
→6)-α-GlcN^I^-(1→***P***	-1.44
→6)-β-GlcN^II^4***P***-(1→	0.48
→3)-α-Hep^I^2*P*4***P***-(1→	0.28
→3)-α-Hep^I^2*P*4***P***-(1→	1.57
→3)-α-Hep^II^6***P***-(1→	1.70

**Table 6:** Mass spectrometric analysis of core-OS of *Pst* DC3000 Δ*wbpL* and wild type. Summary of calculated and observed monoisotopic neutral masses [Da] is given. Accuracy of the measurement is stated as Δppm; n.d. = not detected; * detected, but only with <5% of relative intensity to the major base peak.

Molecule		*Pst* DC3000 Δ*wbpL*		*Pst* DC3000 WT	
	Calculated Mass [Da]	Observed Mass [Da]	Error [Δppm]	Observed Mass [Da]	Error [Δppm]
Kdo_1_Hep_1_HepCm_1_Hex_2_HexN_2_Ala_1_Ac_1_P_3_–*H_2_O*	1646.381	1646.383 *	1.2	1646.384	1.8
**Kdo_1_Hep_1_HepCm_1_Hex_2_HexN_2_Ala_1_Ac_1_P_3_**	1664.390	1664.394 *	2.4	1664.394	2.4
Kdo_1_Hep_1_HepCm_1_Hex_2_6dHex_1_HexN_1_Ala_1_Ac_2_P_3_–*H_2_O*	1673.380	1673.383 *	1.8	1673.384	2.4
**Kdo_1_Hep_1_HepCm_1_Hex_2_6dHex_1_HexN_1_Ala_1_Ac_2_P_3_**	1691.391	1691.393	1.2	1691.395	2.4
Kdo_1_Hep_1_HepCm_1_Hex_2_HexN_2_Ala_1_Ac_1_P_4_–*H_2_O*	1726.347	1726.349	1.2	1726.350	1.7
**Kdo_1_Hep_1_HepCm_1_Hex_2_6dHex_1_HexN_1_Ala_1_Ac_1_P_4_**	1729.347	1729.348	0.6	n.d.	-
**Kdo_1_Hep_1_HepCm_1_Hex_2_HexN_2_Ala_1_Ac_1_P_4_**	1744.358	1744.360	1.1	1744.361	1.7
**Kdo_1_Hep_1_HepCm_1_Hex_2_6dHex_1_HexN_1_Ala_1_Ac_2_P_4_**	1771.357	1771.359	1.1	1771.361	2.3
Kdo_1_Hep_1_HepCm_1_Hex_2_6dHex_1_HexN_2_Ala_1_Ac_3_P_2_–*H_2_O*	1796.494	n.d.	-	1796.497	1.7
**Kdo_1_Hep_1_HepCm_1_Hex_2_6dHex_1_HexN_2_Ala_1_Ac_3_P_2_ (M_2P_)**	1814.504	1814.509 *	2.8	1814.508	2.2
Kdo_1_Hep_1_HepCm_1_Hex_2_6dHex_1_ HexN_2_Ala_1_Ac_2_P_3–_*H_2_O*	1834.449	1834.452 *	1.6	1834.453	2.2
**Kdo_1_Hep_1_HepCm_1_Hex_2_6dHex_1_HexN_1_Ala_1_Ac_2_P_5_**	1851.324	1851.326	1.1	n.d.	-
**Kdo_1_Hep_1_HepCm_1_Hex_2_6dHex_1_HexN_2_Ala_1_Ac_2_P_3_**	1852.460	1852.462	1.1	1852.463	1.6
Kdo_1_Hep_1_HepCm_1_Hex_2_6dHex_1_ HexN_2_Ala_1_Ac_3_P_3_–*H_2_O*	1876.460	1876.462	1.1	1876.463	1.6
**Kdo_1_Hep_1_HepCm_1_Hex_2_6dHex_1_HexN_2_Ala_1_Ac_3_P_3_ (M_3P_)**	1894.470	1894.472	1.1	1894.474	2.1
Kdo_1_Hep_1_HepCm_1_Hex_2_6dHex_1_ HexN_2_Ala_1_Ac_2_P_4_–*H_2_O*	1914.416	1914.418	1.0	1914.419	1.6
**Kdo_1_Hep_1_HepCm_1_Hex_2_6dHex_1_HexN_2_Ala_1_Ac_2_P_4_**	1932.426	1932.428	1.0	1932.430	2.1
Kdo_1_Hep_1_HepCm_1_Hex_2_6dHex_1_ HexN_2_Ala_1_Ac_3_P_4_–*H_2_O*	1956.426	1956.428	1.0	1956.429	1.5
**Kdo_1_Hep_1_HepCm_1_Hex_2_6dHex_1_HexN_2_Ala_1_Ac_3_P_4_ (M_4P_)**	1974.437	1974.439	1.0	1974.440	1.5
Kdo_1_Hep_1_HepCm_1_Hex_2_6dHex_1_ HexN_2_Ala_1_Ac_2_P_5_–*H_2_O*	1994.382	1994.384	1.0	1994.384	1.0
**Kdo_1_Hep_1_HepCm_1_Hex_2_6dHex_1_HexN_2_Ala_1_Ac_2_P_5_**	2012.393	2012.395	1.0	2012.392	−0.5
Kdo_1_Hep_1_HepCm_1_Hex_2_6dHex_1_ HexN_2_Ala_1_Ac_3_P_5_–*H_2_O*	2036.393	2036.395	1.0	2036.396	1.5
**Kdo_1_Hep_1_HepCm_1_Hex_2_6dHex_1_HexN_2_Ala_1_Ac_3_P_5_ (M_5P_)**	2054.403	2054.405	1.0	2054.406	1.5
Kdo_1_Hep_1_HepCm_1_Hex_2_6dHex_1_HexN_2_Ala_1_Ac_2_P_6_–*H_2_O*	2074.348	n.d.	-	2074.343	−2.4
**Kdo_1_Hep_1_HepCm_1_Hex_2_6dHex_1_HexN_2_Ala_1_Ac_2_P_6_**	2092.359	2092.360	0.5	2092.353	−3.3
Kdo_1_Hep_1_HepCm_1_Hex_2_6dHex_1_ HexN_2_Ala_1_Ac_3_P_6_–*H_2_O*	2116.359	2116.361	0.9	n.d.	-
**Kdo_1_Hep_1_HepCm_1_Hex_2_6dHex_1_HexN_2_Ala_1_Ac_3_P_6_ (M_6P_)**	2134.369	2134.371	0.9	n.d.	-

## Data Availability

All data supporting the findings of this study are provided in the manuscript and its Supplementary Files. Additional data supporting the findings of this study are available from the corresponding authors upon request.

## Supplementary Materials

The following are available online at https://www.mdpi.com/1422-0067/22/6/3250/s1.
