# Supplementary material for: Analysis of the Structure and Biosynthesis of the Lipopolysaccharide Core Oligosaccharide of Pseudomonas syringae pv. tomato DC3000
Source: Int J Mol Sci. 2021 Mar 23;22(6):3250. doi: 10.3390/ijms22063250 (PMC8005017; doi:10.3390/ijms22063250)
Supplement: Supplementary file 1 [file ijms-22-03250-s001.zip › ijms-1113669_Supp Info_Rev2.docx]

**Supporting Information**

**Analysis of the structure and biosynthesis of the lipopolysaccharide core oligosaccharide of *Pseudomonas syringae* pv. *tomato* DC3000**

Alexander Kutschera ^1^, Ursula Schombel ^2^, Dominik Schwudke ^2,3,4^, Stefanie Ranf ^1,^* and Nicolas Gisch ^2,^*

^1^ Chair of Phytopathology, TUM School of Life Sciences Weihenstephan, Technical University of Munich, 85354 Freising-Weihenstephan, Germany; alexander.kutschera@tum.de (A.K.), ranf@wzw.tum.de (S.R.)

^2^ Division of Bioanalytical Chemistry, Priority Area Infections, Research Center Borstel, Leibniz Lung Center, Parkallee 1-40, 23845 Borstel, Germany; uschombel@fz-borstel.de (U.S.), dschwudke@fz-borstel.de (D.S.), ngisch@fz-borstel.de (N.G.)

^3^ German Center for Infection Research (DZIF), 23845 Borstel, Germany

^4^ Airway Research Center North, Member of the German Center for Lung Research (DZL), ARCN site: Borstel, Germany

* Correspondence: ngisch@fz-borstel.de; Tel.: +49-4537-188-7190; ranf@wzw.tum.de; Tel.: +49-8161-71-5626

**Materials included**

**Figure S1**: Representative analytical HPAEC-chromatogram for the mixture of oligosaccharides obtained after hydrazinolysis and alkaline hydrolysis (OS-HyKOH) of LPS from *Pst* DC3000 Δ*wbpL*

**Table S1**: Predicted proteomes used for BLASTP experiments.

**Figure S1**

**
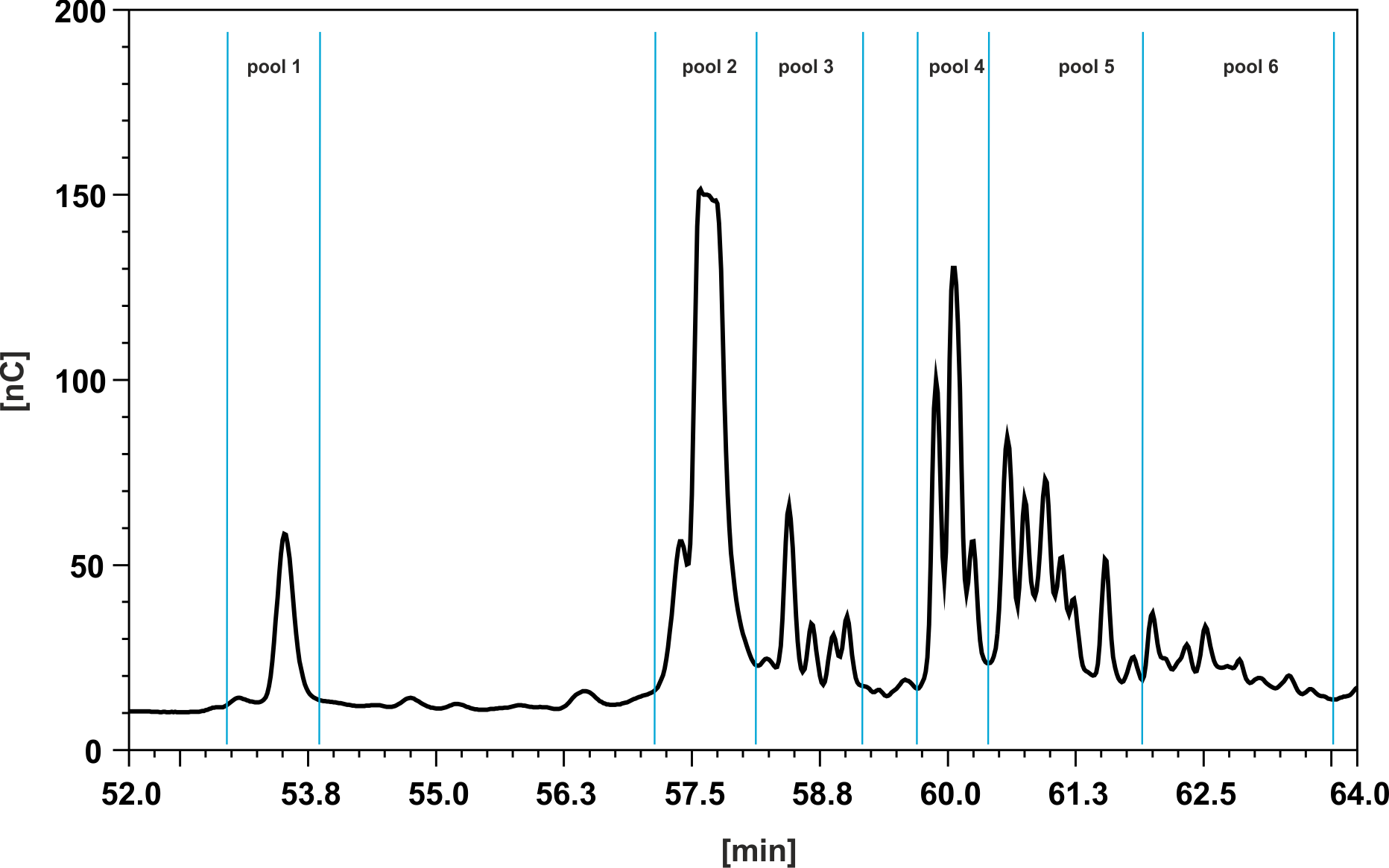
**

**Figure S1. Representative analytical HPAEC-chromatogram for the mixture of oligosaccharides obtained after hydrazinolysis and alkaline hydrolysis (OS-HyKOH) of LPS from *Pst* DC3000 Δ*wbpL*.** Shown is the relevant section (52.0 – 64.0 min), in which the majority of OS-HyKOH molecules is eluting. Assignment of pools is based on the re-analysis of fractions from the preparative separation, which cannot be monitored itself due to neutral pH (for details see Materials & Methods). Pools contained as main components the following molecules (compare Table 2): pool 1: **6**; pool 2: **1**; pool 3: **1**, **5**; pool 4: **3**, **4**, **9**; pool 5: **2**; pool 6: **8**, **2** + *P* (M_calc_: 2516.457 Da; M_obs_: 2516.458 Da; was not detectable in crude mixture).

**Table S1:** **Predicted proteomes used for BLASTP experiments.**

| Organism | UniProt proteome ID | Reference proteome |
| --- | --- | --- |
| *Pseudomonas aeruginosa* PAO1 | UP000002438 | yes |
| *Pseudomonas aeruginosa* PA14 | UP000000653 |  |
| *Pseudomonas cannabina* ICMP2823 | UP000050564 |  |
| *Pseudomonas cichorii* JBC1 | UP000019031 | yes |
| *Pseudomonas fluorescens* Pf0-1 | UP000002704 |  |
| *Pseudomonas fluorescens* Pf-5 | UP000008540 | yes |
| *Pseudomonas fuscovaginae* IRRI 6609 | UP000037931 | yes |
| *Pseudomonas protegens* Cab57 | UP000031621 | yes |
| *Pseudomonas pudita* KT2440 | UP000000556 | yes |
| *Pseudomonas syringae* CC1557 | UP000019089 |  |
| *Pseudomonas syringae* GAW0119 | UP000028631 | yes |
| *Pseudomonas syringae* pv. *japonica* M301072 | UP000004471 | yes |
| *Pseudomonas syringae* pv. *maculicola* ES4326 | UP000003811 |  |
| *Pseudomonas syringae* pv. *maculicola* M4a | UP000037879 |  |
| *Pseudomonas syringae* pv. *oryzae* 1_6 | UP000005313 |  |
| *Pseudomonas syringae* pv. *phaseolicola* 1448a | UP000000551 |  |
| *Pseudomonas syringae* pv. *syringae* B728a | UP000000426 |  |
| *Pseudomonas syringae* pv. *tagetis* ICMP 4091 | UP000050474 |  |
| *Pseudomonas syringae* pv. *tomato* DC3000 | UP000002515 | yes |
